# Phytochemical screening, antioxidant activity of selected methanolic plant extracts and their detoxification capabilities against AFB_1_ toxicity

**DOI:** 10.1016/j.heliyon.2024.e24435

**Published:** 2024-01-11

**Authors:** Mavie Rose Kongolo Kalemba, Rhulani Makhuvele, Patrick Berka Njobeh

**Affiliations:** a Department of Biotechnology and Food Technology, Faculty of Science, University of Johannesburg, Doornfontein Campus, P.O. Box 17011, Gauteng, 2028, South Africa; b Toxicology and Ethnoveterinary Medicine, ARC-Onderstepoort Veterinary Research, Private Bag X05, Onderstepoort, 0110, South Africa

**Keywords:** Aflatoxin B_1_, Antioxidants, Detoxification, Phytochemicals, And reactive oxygen species

## Abstract

Aflatoxin B_1_ (AFB_1_) is a secondary metabolite produced principally by *Aspergillus parasiticus* and *A. flavus*. It is one of the most potent and commonly occurring dietary carcinogen with its carcinogenic potential being linked to the formation of DNA adducts and reactive oxygen species (ROS). Plant extracts contain a plethora of biologically active phytochemicals that act against ROS. This study aimed to assess the phytochemical content and antioxidant activity of methanolic extracts of some medicinal plants and investigate their detoxification potentials against AFB_1_. Phytochemical screening together with total phenolic content (TPC), total flavonoid content (TFC), and antioxidant (2,2-diphenyl-1-picrylhydrazyl (DPPH) and 2,2-azino-bis-(3-ethylbenzothiazoline-6-sulfonic acid) diammonium salt (ABTS^+^)) assays) were performed on nine methanolic plant extracts. Extracts were incubated with AFB_1_ for 24 and 48 h and liquid chromatography mass spectrometry (LC-MS) analysis done to assess their AFB_1_ detoxification activities. The TPC of the extracts ranged from 88.92 ± 6.54 to 210.19 ± 7.90 mg GAE/g, while TFC ranged between 4.01 ± 0.94 and 32.48 ± 1.02 mg QE/g. Radical scavenging activities of extracts varied from 4.18 ± 1.37 to 251.53 ± 9.30 μg/mL and 8.36 ± 1.65 to 279.22 ± 8.33 μg/mL based on DPPH and ABTS^+^ assays, respectively. Six of the plant extracts showed a time-dependent detoxification activity against AFB_1_ after 48 h ranging from 20.17 to 38.13 %. *C. dentata* bark extract showed the highest percentage of AFB_1_ reduction, with mean percentages of 43.57 and 70.96 % at 24 and 48 h, respectively. This was followed by *C. asiatica* leaves and *A. melegueta* seeds with a maximum of 40.81 and 38.13 %, respectively after 48 h. These extracts also possessed high TPC, TFC, and antioxidant activities compared to all the other extracts. Findings from this study demonstrate the abundance of bioactive compounds with antioxidant activity playing a role in potent AFB_1_ detoxification activity.

## 1 Introduction

The presence of mycotoxins, particularly AFB_1_ in food and feed at certain risk levels can threaten the well-being of humans and animals [1]. AFB_1_ is a genotoxin and hepatocarcinogen that is believed to cause cancer by generating DNA adducts in target cells, thus causing genetic changes, oxidative damage, DNA strand breakage, and DNA base damage [2]. Metabolic conversion of AFB_1_ by the cytochrome p450 system to guanine binding AFBO explained the toxicology of AFB_1_ at a molecular level. However, research also suggests that the immunotoxic impact produced by mycotoxins including AFB_1_ may be linked to the increased production of ROS during metabolism thus causing oxidative stress which result in cell damage by protein oxidation, depletion of thiols, and lipid peroxide [3,4]. Therefore, the development of reliable techniques to eliminate AFB_1_ contamination of foods and other commodities is important.

Plants have been utilized since the beginning of human existence to cure numerous diseases as they contain antioxidants, antitumor, anticancer, antimicrobial, antimutagenic, and hepatoprotective agents [5,6]. The use of plants for the control and degradation of mycotoxins is an interesting avenue to explore [[7], [8], [9], [10], [11]]. Several studies have demonstrated the effect of different parameters on detoxification of AFB_1_ by various medicinal plant extracts [[7], [8], [9]]. Iram et al. [7] studied temperature and time course of AFB_1_ degradation by *Corymbia citriodora.* The results showed that detoxification occurred within 3 h and gradually increased as incubation time increases, with maximum degradation observed after 72 h. They further observed maximum detoxification at 60 °C, however proceeded the experiment at 45 °C to eliminate the effect of heat and moisture on toxin inactivation. Correspondingly, Vijayanandraj et al. [8] explored the detoxification potential of *Adhatoda vasica* leaves at different time intervals of 3, 6, 12, 24 and 48 h at 37 °C. They found that the leaf extract showed maximum AFB_1_ degradation of 98 % after incubating for 24 h. In other studies, by Iram et al. [9] *Trachyspermum ammi* was able to reduce AFB_1_ and B_2_ by 90 and 89 % while Al-Owaisi et al. [11] obtained over 70 % of AFB_1_ detoxification from herbal extracts of *Centella asiatica*, *Eclipta prostrata*, and *Hybanthus enneaspermus*. This literature therefore illustrates that plants contain components that possess degradation/detoxification capabilities against AFB_1_. In addition, exploring natural plant products as a means of detoxifying AFB_1_ maybe the best option not only because they are generally safe, and easily available but that their phytochemical constituents can add to the nutritional content and reduce the effect of ROS that contribute to the toxic impact produced by AFB_1_ as well.

Africa is richly endowed with a wealth of medicinal plants. However, very few studies have investigated their detoxification potentials against mycotoxin [12]. This is of particular importance because the African continent is highly affected by mycotoxin contamination [13,14]. The plant species under investigation were chosen based on their history in traditional medicine and they include *Aframomum melegueta, Centella asiatica, Cheilanthes contracta, Curtisia dentata, Hypoxis hemerocallidea, Nymphaea stellata*, and *Pittosporum viridiflorum.* These plant species are widely used in African traditional medicine for treatment of inflammation and cancer, which are both linked to oxidative stress. These plant species also possess antioxidant, antimicrobial, anti-inflammatory and anticancer properties [[15], [16], [17], [18], [19], [20]] however, little is known about their mycotoxin detoxification capabilities. Thus, the present study aimed to investigate the phytochemical contents and antioxidant activities of selected indigenous plants from Africa and further evaluate their detoxification capabilities against dietary carcinogen AFB_1_.

## 2 Materials and methods

### 2.1 Plant collection and preparation

Different plant part materials of selected plant species (Table 1) were gathered from the South African National Biodiversity Institute (SANBI; Pretoria botanical gardens) in Pretoria, South Africa (RSA), Mbaza Ngunbu district in Kilombu village Democratic Republic of Congo (DRC) and other materials were purchased at the Faraday Muthi market, Johannesburg (RSA) in summer (March 2021). The identities of the plants collected at SANBI were verified by Mr N. Hlongwane from SANBI, Pretoria. The voucher specimens were deposited at the University of Johannesburg Herbarium (JRAU). The plant materials were rinsed with distilled water to remove excess soil debris and dried by open air exposure in the lab away from direct sunlight for 2 weeks. Thereafter, the plant materials were ground to powder, then kept in airtight glass containers and stored at room temperature away from sunlight.

**Table 1:** Table showing the scientific names, voucher specimen numbers, common names, and the plant parts used to conduct this study.

Scientific name	Common name/s	Plant part used	Location	Voucher specimen no.
***A. melegueta***	Grains of paradise, alligator pepper (Eng.), mudongo (Lin.)	Seeds	Mbaza Ngunbu district in Kilombu village	M.R. Kongolo Kalemba and K.C. KiKalulu 4
***C. asiatica***	Indian pennywort (Eng.)	Leaves	Pretoria National Botanical Garden	M.R. Kongolo Kalemba and R. Makhuvele 1
***C. contracta***	Parsley fern (Eng.), lehorometso (S), inkomakoma (Z)	Rhizomes	Faraday Muthi Market	M.R. Kongolo Kalemba 6
***C. dentata***	Assegai (Eng.), umLahleni (X, Z), assegaai (Afr.)	Bark	Faraday Muthi Market	M.R. Kongolo Kalemba 5
***H. hemerocallidea***	African potato (Eng.), sterretjie (Afri.), inkomfe (Z)	Leaves	Pretoria National Botanical Garden	M.R. Kongolo Kalemba and R. Makhuvele 2
***N. stellate***	Water lily (Eng.)	Stem, flowers, and leaves	Pretoria National Botanical Garden	M.R. Kongolo Kalemba and R. Makhuvele 3
***P. viridiflorum***	Cheesewood (Eng.), umfasamvu (Z), umkhwenkwe (X), stinkbas (Afri.)	Bark	Faraday Muthi Market	M.R. Kongolo Kalemba 7

Notation: Afri. - Afrikaans, Eng.- English, Lin.- Lingala, *S*- Sotho, X- Xhosa, Z- Zulu.

### 2.2 Plant extraction

The plant materials were extracted using 80 % methanol (Merck, Darmstadt, Germany) at a ratio of 1:10 plant material to solvent by maceration. Ten grams of the plant material were dissolved in 100 mL of methanol in Schott bottles. The mixture was then allowed to stand for 24 h and occasionally shaken. Thereafter, the extracts were separated by filtration using Whatman No. 4 filter paper (Whatman Inc., Florham Park, U.S.A). The resultant filtrate was concentrated using a rotary evaporator (EV311PLUS, LabTech, China) at 35 °C. The extract was transferred into concentrator at 30 °C and concentrated to dryness using a vacuum concentrator (Concentrator plus, Eppendorf, U.S.A) and thereafter, transferred into vials. The percentage extract yield of the extracts was expressed by dividing the total mass extracted after concentrating by the mass of the dried plant sample used for extraction. Stock solutions of 10 mg/mL were prepared for each extract.

### 2.3 Phytochemical screening

#### 2.3.1 Qualitative phytochemical screening

The identification of phytochemical contents of the methanolic plant extracts was carried out using standard phytochemical screening methods. Anthraquinones, flavonoids, terpenoids, quinones, tannins, phenols, cardiac glycosides, and saponins, were screened [21].

#### 2.3.2 Phytochemical screening using thin layer chromatography plate

The thin layer chromatography (TLC) method was employed to separate the plant extract and evaluate their constituents. Ten microliter of each plant extract (1 mg/mL) was spotted on TLC plates (Whatman Inc., Florham Park, U.S.A) and were placed in TLC chambers containing different mobile phases with different polarities, namely EMW (ethyl acetate (Sigma Aldrich, Darmstadt, Germany)/methanol/water (40:5.4:4), CEF (chloroform (Merck, Darmstadt, Germany)/ethyl acetate (Sigma Aldrich, Darmstadt, Germany)/formic acid (Sigma Aldrich, Darmstadt, Germany) (5:4:1, v/v/v), and BEA (benzene (Sigma Aldrich, Darmstadt, Germany)/ethanol (Sigma Aldrich, Darmstadt, Germany)/ammonium hydroxide (Merck, Darmstadt, Germany) (90:10:1, v/v/v). The plates were developed and thereafter, visualized under UV light at wavelengths of 254 nm and 365 nm. The plates were sprayed with *p*-anisaldehyde-sulphuric acid (Sigma Aldrich, Darmstadt, Germany) and heated at 100 °C to allow color development to determine the phytochemical fingerprints of the extracts.

### 2.4 Quantitative phytochemical screening

#### 2.4.1 Determination of total phenolic content (TPC) assay

The total phenolic content of the different crude extracts was assessed spectrophotometrically using the Folin ciocalteu assay [22]. Five hundred microliter of the freshly prepared 10× diluted Folin-Ciocalteu reagent (Protea Lab, Johannesburg, South Africa) was mixed with an aliquot of 100 μl of the plant extract (1 mg/mL) and left to stand for 5 min at room temperature. To neutralize the mixture, 500 μl of sodium bicarbonate solution (Sigma Aldrich, Darmstadt, Germany) (7.5 %, w/v) was added and the resulting mixture was kept in the dark at room temperature for 30 min. Following incubation, the absorbance of the solution was measured at 765 nm using UV–vis spectrophotometer (Hexiose, Thermo Spectronic, U.S.A). Gallic acid (SRL, Maharashtra, India) (0–200 μg/mL) was used as a reference standard and the standard curve was constructed.

#### 2.4.2 Determination of total flavonoid content (TFC)

The total flavonoid content of the extracts was conducted by aluminum chloride colorimetric method according to the procedure adapted from Ref. [22]. Briefly, 100 μl of plant extract (1 mg/mL) was further diluted with 750 μl of 90 % methanol, and then mixed with 50 μl of aluminum chloride (Protea Lab, Johannesburg, South Africa) and 50 μl of 1 M potassium acetate (Sigma Aldrich, Darmstadt, Germany). The mixture was kept for 30 min at room temperature before recording absorbance at 415 nm. Quercetin (Sigma Aldrich, Darmstadt, Germany) (0–100 μg/mL) was used as a reference standard to construct the standard curve.

### 2.5 Antioxidant activities

#### 2.5.1 Qualitative antioxidant activities

TLC method was employed to evaluate the DPPH and ABTS^+^ scavenging activities of the extracts in support of the spectrometric serial dilution approach. A 10 μl of each plant extracts (1 mg/mL) was spotted on TLC plates and developed on different mobile phases with different polarities, EMW (40:5.4:4), CEF (5:4:1, v/v/v) and BEA (90:10:1, v/v/v). Thereafter, the plates were viewed under UV light at wavelengths of 254 nm and 365 nm and sprayed with DPPH and ABTS^+^ solution to detect antioxidant activity of the plant extracts. Clear spots on a purple or blue-green background on a TLC plate sprayed with DPPH (Sigma Aldrich, Darmstadt, Germany) or ABTS^+^ (Sigma Aldrich, Darmstadt, Germany) indicated antioxidant activity.

#### 2.5.2 Quantitative antioxidant activities

##### **2.5.2.1** 2, 2-diphenyl-2-picryl-hydraxyl (DPPH) radical scavenging assay

The free radical-scavenging activity of the plant extracts was determined according to the method adopted by Mongalo et al. [23] with slight modification. Different concentrations of the plant extracts were prepared by serial dilution (1.95–1000 μg/mL). Five hundred microliters of the 0.2 % DPPH solution were mixed with 500 μl of the serially diluted plant extracts. The tubes were incubated for 30 min at 37 °C in the dark and absorbance was measured at 517 nm. DPPH and methanol were used as the blank control, ascorbic acid (Sigma Aldrich, Darmstadt, Germany) and TROLOX (Sigma Aldrich, Darmstadt, Germany) were used as the positive control at the same concentrations as the extracts. All tests were performed in triplicate. The capability to scavenge the DPPH radical was calculated using equation (1).

(1) $$\text{DPPH}\;\text{Scavenged}\;\left(\%\right)=\left(\left(A_{B\;}-\;A_{s}\right)/A_{B}\right)\times 100$$

Where A_B_ is the absorbance of the blank solution and A_S_ is DPPH radical + plant extract. The results were expressed as minimum Inhibitory concentration (IC_50_).

##### **2.5.2.2** 2, 2′-azino-bis (3-ethylbenzothiazoline-6-sulfonic acid) (ABTS^+^) radical scavenging assay

The ABTS radical cation decolorization assay was performed according to Hussen & Endalew [24], with slight modifications. The ABTS^+^ solution (Sisco, Mumbai, India) was prepared at a 1:1 ratio by mixing 2.45 mM potassium persulfate ((Sigma Aldrich, Darmstadt, Germany) with 7 mM ABTS^+^ in distilled water. Prior to use, the solution was stored at room temperature for 16 h in the dark. The resulting solution was further diluted with 90 % methanol to obtain an absorbance of 0.700 at 734 nm, this was used as the working solution. The plant extracts and positive controls were prepared like those of the DPPH radical scavenging assay by mixing 500 μl ABTS^+^ with 500 μl of sample. Thereafter, incubating for 5 min at 37 °C in the dark before taking absorbance readings at 734 nm. Percentage inhibition was calculated using equation (2).

(2) $${\text{ABTS}}^{+}\;\text{Scavenged}\;\left(\%\right)=\left(\left(\text{AB}\;-\;\text{As}\right)/\text{AB}\right)\times 100$$

Where AB is the absorbance of the blank solution and A_S_ is ABTS radical + plant extract. The results were expressed as minimum inhibitory concentration (IC_50_).

### 2.6 AFB_1_ detoxification assay by selected plant extracts

The AFB_1_ detoxification assay was conducted according to the protocol described by Iram et al. [9]. The samples were prepared by mixing 500 μl of plant extract (10 mg/mL) with 100 μl AFB_1_ (Sigma Aldrich, Darmstadt, Germany) (100 ng/mL) dissolved in acetonitrile (Merck, Darmstadt, Germany). The control sample constituted of 100 μl AFB_1_ solution with 500 μl of methanol. The final concentration of AFB_1_ in the mixtures or controls was 17.31 ng/mL. The resulting mixtures were incubated at 37 °C for 24 and 48 h. After incubation, 500 μl of chloroform was added to the mixture to stop the reaction. This was followed by 1 min of low centrifugation. The samples were then evaporated and reconstituted with methanol then filtered through a 0.22 μm PVDF syringe filter (Merck, Darmstadt, Germany) and analyzed on the liquid chromatography with tandem mass spectrometry (LC-MS) 8040 system (Shimadzu, U.S.A). An internal calibration curve was used to quantify the amount of AFB_1_ in the test samples. This was achieved by utilizing AFB_1_ standard dissolved in acetonitrile at various concentrations (1.56, 3.13, 6.25, 12.5, 25, 50, 100 ng/mL). The calibration curve was acquired by linking the peak areas of the AFB_1_ standard with their various concentrations.

#### 2.6.1 LC-MS analysis

The LC-MS 8040 system was used to quantify the amount of AFB_1_ in the test samples. The mobile phase consisted of methanol: acetonitrile: water (22.5:22.5:55.0; v/v/v). A raptor column (Raptor ARC-18 column of 2.1 × 100 mm, 2.7 μm particle size) kept at 40 °C was used for separation in isocratic mode. The injection volume of each sample was 10 μl with a flow rate of 0.5 mL/min. All analysis was performed in triplicate. The MS conditions were as follows: capillary temperature was 335 °C, sheath gas flow and auxiliary gas flow were 20 and 4 L/min, respectively. Source voltage, capillary voltage, and tube lens voltage were 5 KV, 49 V, and 120 V, respectively.

The identification of AFB_1_ was aided by the monitoring of two minimum product ions (1 quantifier and 1 qualifier). The triple quadrupole (TQD) mass spectrometer was used to detect the analyte through an MS/MS system. The analyte was delivered to the MS through electron spray ionization (ESI). The ESI was kept in a positive mode with the following settings: 250 °C desolation line (DL), 400 °C source block heater, 3 L/min nebulizing interface gas flow, and 15 mL/min drying gas flow. The data collecting system, which utilized multiple reaction monitoring (MRM), was adapted to fit the analyte MS parameters.

### 2.7 Statistical analysis

The statistical analysis software IBM SPSS statistics 27 was used to obtain the data presented in the study. The experiment was done in triplicates and the data is presented as mean ± standard deviation (SD). The one-way ANOVA statistical analysis was used to determine the significant differences between the mean values were probability of P < 0.05 was considered as significant.

## 3 Results

### 3.1 Phytochemical screening

**Fig. 1:**
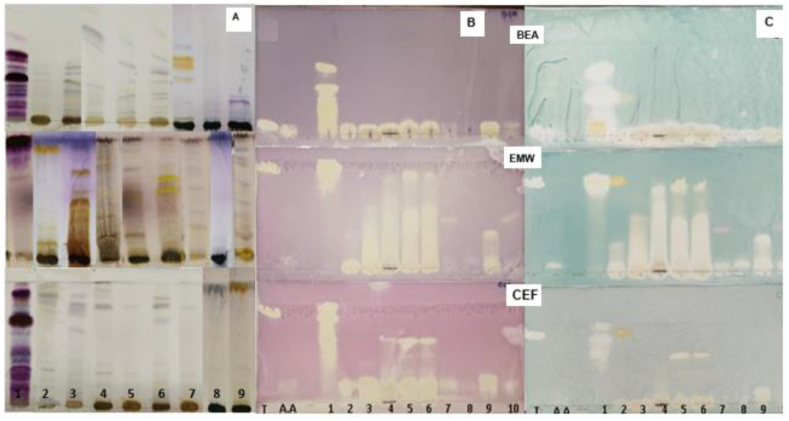
TLC chromatogram of nine plant extracts developed on a mobile phase: benzene: ethanol: ammonium hydroxide (BEA), ethyl acetate: methanol: water (EMW) and chloroform: ethyl acetate: formic acid (CEF); and sprayed with (a) *p*-anisaldehyde-sulphuric acid, (b) 0.2 % DPPH and (c) ABTS^+^ to show the presence of antioxidants compounds (yellow to clear zones). Notation: T-Trolox; A. A - ascorbic acid; 1- *A. melegueta*; 2- *C. contracta*; 3- *C. dentata*; 4. *H- hemerocallidea*; 5–7. *N. stellata* (flowers, leaves, and stems); 8- *P. viridiflorum*; 9- *C. asiatica*.

The results of the phytochemical analysis of the crude methanolic extracts of the various plant species are reported in Table 2. It was discovered that all the extracts contained flavonoids and phenols. Only *A. melegueta* and *C. dentata* extracts indicated the presence of anthraquinones. Apart from *P. viridiflorum*, tannins were found in all extracts. Complex terpenoids were observed in all extracts, except for *A. melegueta, N. stellata stems,* and *C. contracta*, contained structurally. Only extracts of *C. dentata*, *C. contracta*, and *P. viridiflorum* were found to have quinones. Flowers of *H. hemerocallidea* and *N. stellata* showed the presence of cardiac glycosides. Lastly, *A. melegueta* was the only extract that tested negative for saponins. These groups of phytochemicals present in plant extracts might be responsible for their pharmacological activity. The phytochemical profiling using TLC showed the presence of different plant compounds, which include polar, non-polar, and intermediate compounds in all three mobile phases used (Fig. 1a). However, *P. viridiflorum* showed less compounds as evident by the presence of few bands in all mobile phases.

**Table 2:** Phytochemical constituents of selected methanolic crude plant extracts.

**Plants species**	**Extraction yields (% w/w)**	**Plant part used**	**Anthraquinones**	**Flavonoids**	**Terpenoids**	**Quinones**	**Tannins**	**Phenols**	**Cardiac glycosides**	**Saponins**
***A. melegueta***	35.26	Seeds	+	+	–	–	+	+	–	–
***C. asiatica***	79.35	Leaves	–	+	+	–	+	+	–	+
***C. contracta***	33.68	Rhizomes	–	+	–	+	+	+	–	+
***C. dentata***	70.09	Bark	+	+	+	+	+	+	–	+
***H. hemerocallidea***	54.26	Leaves	–	+	+	–	+	+	+	+
***N. stellata***	62.40	Leaves	–	+	+	–	+	+	–	+
	51.97	Flowers	–	+	+	–	+	+	+	+
	14.81	Stems	–	+	–	–	+	+	–	+
***P. viridiflorum***	20.68	Bark	–	+	+	+	–	+	–	+

Notation:= Absence of phytochemical, + = Presence of phytochemical.

### 3.2 Total phenolic content

The total phenolic content of the extracts was determined from the standard curve of gallic acid with the equation y = 0.004x + 0.0503, with R^2^ = 0.9881. As indicated in Table 3, the phenolic content was expressed as gallic acid equivalents (GAE) per gram of dry weight. The extracts had phenolic concentrations ranging from 88.92 mg GAE/g to 210.19 mg GAE/g. *C. dentata, A. melegueta, N. stellata* leaves and flowers possessed significant high phenolic contents when compared with *P. viridiflorum* (P ≤ 0.05).

**Table 3:** Radical scavenging capacity of nine plant extracts and their total phenolic and flavonoid contents. The values are expressed as the mean ± standard error (n = 3).

Extracts	**DPPH (IC**_**50**_**μg/mL)**	**ABTS**^**+**^**(IC**_**50**_**μg/mL)**	TPC GAE (mg/g)	TFC QE (mg/g)
***A. melegueta***	4.18^a^ ± 1.37	8.36^ab^ ± 1.65	178.19^e^ ± 2.37	32.48^g^ ± 1.02
***C. asiatica***	18.16^de^ ± 3.33	20.62^c^ ± 2.61	133.73^d^ ± 5.08	16.73^e^ ± 2.20
***C. contracta***	22.70^e^ ± 2.37	30.21^d^ ± 2.49	122.39^c^ ± 5.87	8.86^cd^ ± 1.13
***C. dentata***	10.34^abc^ ± 1.43	9.01^ab^ ± 1.36	210.19^f^ ± 7.90	15.90^e^± 1.13
***H. hemerocallidea***	14.69^bcd^ ± 2.79	19.99^c^ ± 2.27	127.71^cd^ ± 2.96	25.33^f^ ± 1.30
***N. stellata* flowers**	7.29^a^ ± 2.20	11.42^ab^ ± 2.29	169.79^e^ ± 6.02	24.37^f^ ± 2.42
***N. stellata* leaves**	8.90^ab^ ± 3.14	13.02^b^ ± 0.70	174.39^e^ ± 8.40	11.51^d^ ± 1.41
***N. stellata* stem**	52.55^f^ ± 4.91	60.18^e^ ± 7.19	119.66^c^ ± 8.10	5.56^c^ ± 1.17
***P. viridiflorum***	251.53^g^ ± 9.30	279.22^f^ ± 8.33	88.92^b^ ± 6.54	4.01^b^ ± 0.94
**Ascorbic acid**	17.06^cde^ ± 0.70	10.34^ab^ ± 2.13	–	–
**Trolox**	14.93^bcd^ ± 2.00	5.74^a^± 2.10	–	–

Values in the same column followed by a different letter (^a-f^) are significantly different (*P* < 0.05).

### 3.3 Total flavonoid content

As shown in Table 3, the estimated flavonoid content of the plant extracts was represented in terms of mg/g quercetin equivalent (QE). The quercetin standard curve's linear equation, of y = 0.0217× - 0.0137 with R^2^ = 0.9957, was used to determine the TFC. The TFC ranged from 4.01 to 32.48 QE (mg/g). *A. melegueta, H. hemerocallidea, N. stellata* flowers, *C. asiatica, C. dentata, and N. stellata* leaves possessed significant flavonoid contents when compared with *P. viridiflorum* (P ≤ 0.05). No significant difference (P > 0.05) was observed between the TPC values of *N. stellata* flowers and leaves.

### 3.4 Qualitative antioxidant activities using TLC bioautography

For qualitative antioxidant activities using TLC bioautography, yellow to clear zones against the purple or blue-green background indicated the presence of antioxidant compounds (Fig. 1 b and c)*. A. melegueta*, *H. hemerocallidea*, *C. dentata*, *N. stellata* (flowers and leaves), *C. asiatica*, and *C. contracta* had considerable activity, whereas those of *P. viridiflorum* and *N. stellata* stems demonstrated weak activity in both DPPH and ABTS ^+^ assays. EMW plates had more antioxidants compounds followed by CEF and lastly BEA in all assays.

### 3.5 Quantitative antioxidant assay (DPPH and ABTS^+^)

**Fig. 2:**
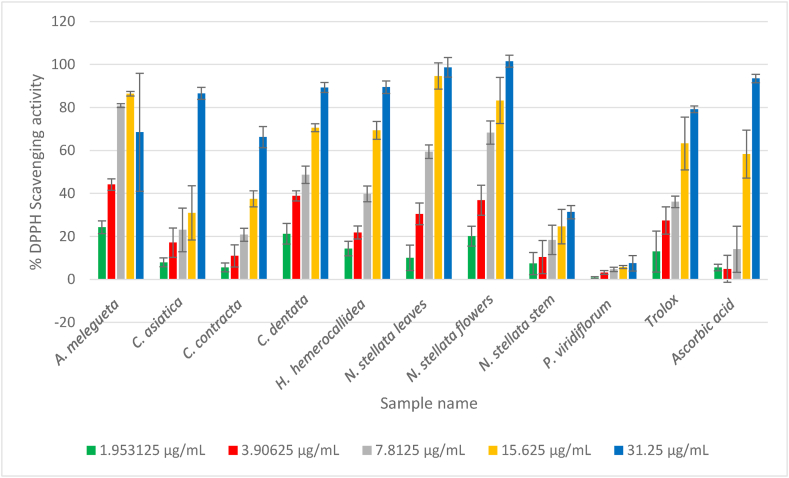
DPPH radical scavenging activity (%) versus concentration (μg/mL).

**Fig. 3:**
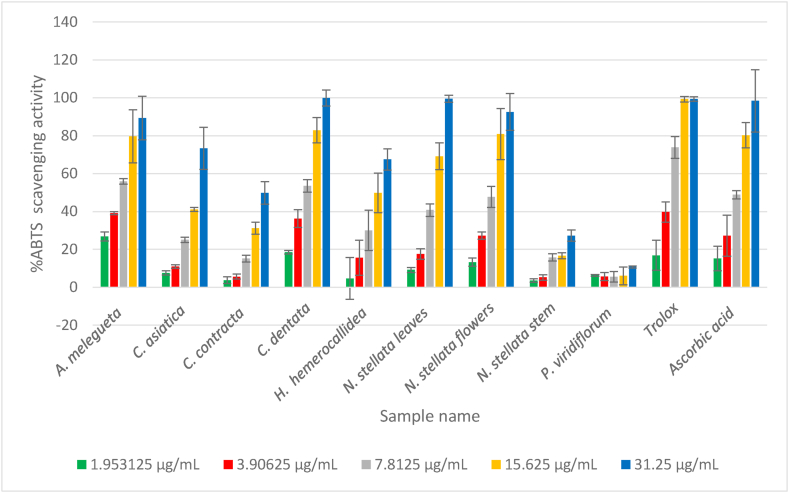
ABTS radical scavenging activity (%) versus concentration (μg/mL).

The IC_50_ values were calculated as the concentration of plant extracts that could scavenge 50 % of the free radicals (Table 3). Fig. 2, Fig. 3 show the radical scavenging (%) activities of the plant extracts against DPPH and ABST radicals, respectively. Lower IC_50_ values indicate stronger antioxidant activity while high IC_50_ values indicate weak antioxidant activities. The antioxidant activities of the plant extracts varied from 4.18 to 251.53 μg/mL and 8.36–279.22 μg/mL for DPPH and ABTS^+^ assays, respectively. The results were compared to well-known antioxidant standards TROLOX and ascorbic acid. All tested plant extracts had strong antioxidant activities except for *P. viridiflorum* showed significant weak antioxidant activities (P ≤ 0.05) in both antioxidant assays*. A. melegueta, C. dentata, N. stellata* flowers and leaves had stronger antioxidant activities when compared with ascorbic acid (17.06 μg/mL) and TROLOX (14.93 μg/mL) in DPPH assay, while *A. melegueta* showed strongest antioxidant compared to ascorbic acids. These results are in line with the results obtained from qualitative antioxidant activities using TLC (Fig. 1).

### 3.6 Detoxification of AFB_1_ by plant extracts

Methanolic plant extracts were assessed for their detoxification capability against AFB_1_ at different intervals (Table 4). A time-dependent reduction in AFB_1_ level was observed in both the control and the tested plant extracts. The control sample showed 54.24 and 57.82 % AFB_1_ reduction after 24- and 48-h incubation, respectively. The detoxification capacity of the extracts was estimated by comparing AFB_1_ concentrations in the plant extracts to the respective control incubated under the same conditions. The highest percentage (71.13 %) of AFB_1_ reduction was observed in *C. dentata* bark extract at 48 h. Extracts of *C. asiatica* and *A. melegueta* also showed notable detoxification activity with maximum of 40.84 % and 37.86 % after 48 h, respectively. There was no significant difference (P > 0.05) between the detoxification activity of *N. stellata* flowers (18.54 %) and leaves (19.99 %).

**Table 4:** Concentration of AFB_1_ recovered and percentage reduction of AFB_1_ in the presence of methanolic plant extracts (n = 3).

Treatment	AFB_1_ reduction	
	24 h	48 h
***A. melegueta +* AFB**_**1**_	17.37^b^ ± 10.90	37.86^b^ ± 6.20
***C. asiatica +* AFB**_**1**_	28.39^c^± 0.39	40.84^b^ ± 5.31
***C. contracta +* AFB**_**1**_	nd	nd
***C. dentata +* AFB**_**1**_	44.82^d^ ± 2.59	71.13^d^ ± 4.49
***H. hemerocallidea +* AFB**_**1**_	13.59^b^ ± 9.92	14.62^a^± 10.06
***N. stellata* flowers *+* AFB**_**1**_	14.34^b^ ± 0.60	18.54^a^± 2.16
***N. stellata* leaves *+* AFB**_**1**_	2.69^a^ ± 3.68	19.99^a^± 5.93
***N. stellata* stems *+* AFB**_**1**_	nd	nd
***P. viridiflorum +* AFB**_***1***_	nd	nd
**Control (AFB**_**1**_ + **methanol)**	54.24^d^ ± 0.52	57.82^c^± 2.69

Notation: Nd-means the compound is not determined.

Values in the same column followed by a different letter (^a-f^) are significantly different (P < 0.05).

## 4 Discussion

The phytochemical analysis revealed the presence of various classes of bioactive compounds in all tested methanolic plant extracts in both the qualitative and quantitative methods used in this study. These phytochemicals have shown to exhibit antioxidants, antimalarial, antimicrobial, anti-inflammatory, antitumor and preventative effects against different diseases [[25], [26], [27], [28], [29], [30], [31]]. Structurally complex terpenoids were identified in all extracts except that of *A. melegueta*, *N. stellata* stems, and *C. contracta*. All extracts except *P. viridiflorum* revealed the presence of tannins which are reported to have antibacterial, antitumor, and antiviral activities [25]. Anthraquinones which have previously been reported to have anti-inflammation, anticancer, antihyperlipidemic, and immunoregulation effects [26] was only present in extracts of *A. melegueta* and *C. dentata*. Cardiac glycosides, which are used to treat cardiac arrhythmia and congestive heart failure [27] was found in *H. hemerocallidea* and *N. stellata* flowers. This could support the reason behind the use of *N. stellata* flowers in treatment of heart palpitations in India [28]. Quinones which have been reported to have antitumor activities were only seen in extracts of *C. dentata*, *C. contracta,* and *P. viridiflorum,* these plant parts are used in South African traditional practices for cancer treatment [29]. Lastly, *A. melegueta* was the only extract that tested negative for saponins, which are reported to lower the risk of cancer, blood glucose response, and blood lipid levels [30]. Saponins are also reported to have anti-viral and anti-bacterial properties [31].

Flavonoids and phenols are believed to be the most promising and measured phytochemical constituents among plant secondary metabolites [22,1]. The hydroxyl groups in phenols and flavonoids are important because they provide redox properties critical for antioxidant action [32,33]. The phenolic and flavonoid content demonstrated that C. *dentata* and *A. melegueta* possessed the highest phenolic and flavonoid content, respectively. The TPCs observed in this study for the methanolic extracts of *C. dentata,* A. *melegueta* and *N. stellata* leaves were much higher than those reported by earlier researchers on the same species using various extraction solvents [[32], [34],^35^,^36^]. Earlier studies have reported 14.86 mg/g [35], 160.77 mg GAE/g [36], 6.5 mg GAE/g [32], and 18.40 mg/100 mg [34] for *C. dentata*, *A. melegueta*, *N. stellate* leaves and flowers, respectively. Moreover, Wintola & Afolayan [37] reported highest flavonoid content in ethanol extract which is relatively similar to that of the methanolic extract of *C. dentata* obtained in this study. Based on the results obtained, it can said that flavonoid compounds of *C. dentata* are of a polar nature because flavonoid content determined in the extracts increased as polarity of the extraction solvent increased. Nonetheless, there is no information on phytochemical studies of methanolic leaf extract of *C. contracta* and *H. hemerocallidea*. The whole plant along with rhizome of plants belonging to the genus *Cheilanthes* has shown to contain phenolic compounds [38,39] obtained 204.56 μg/g of phenolics in *H. hemerocallidea* corm extract. The variation between the values obtained in this research for TPC and TFC, and those reported by literature may be due to several factors such as geographical location of the plant species, environmental factors, age of plant, time of harvest, methods of extraction, which include solvent choice and duration. All of these have a profound effect on the bioactive constituents of the plant [40]. Evidently, in a study to investigate the impact of environmental conditions on active substance contents and antioxidant activity of *Potentilla fruticosa* from different regions in China, Liu et al. [41] found that content of the active components in *P. fruticosa* leaves varied greatly depending on where they were cultivated. The investigation further concluded that annual mean temperature significantly and negatively correlated to the TPC, whereas the altitude significantly and positively correlated to the TPC (P < 0.05).

According to the phytochemical screening results, the plant extracts contained a variety of secondary metabolites such as glycosides, flavonoids, tannins, etc., all of which according to Ref. [22], have the potential to discolour both ABTS^+^ and DPPH solutions due to their hydrogen donating ability, indicating potent bioactivity. To varying degrees, all plant extracts displayed antioxidant activity. Plates developed on EMW and CEF revealed the presence of more radical inhibitors after spraying with ABTS^+^ and DPPH as seen in Fig. 1b and c. This therefore suggested that majority of the radical inhibitor compounds present in the extracts were of a polar nature, which was expected as the extraction solvent methanol favours extraction of polar compounds. Furthermore, the antioxidant activities of the plant extracts using spectrometric methods were interpreted using the classification proposed by Ref. [42]. That is, extracts with IC50 values of less than10 μg/mL were deemed extremely strongly active, while those with values ranging from 10 to 50 μg/mL were considered to have strong antioxidant activity, and those with values ranging from 50 to 100 μg/mL were regarded to have moderate antioxidant activity. Extracts having IC50 values greater than 250 μg/mL were considered inactive. DPPH assay (Table 3) revealed that extracts of *A. melegueta* and *N. stellata* flowers and leaves exhibited extremely strong antioxidant activity, with IC_50_ values less than 10 μg/mL. *C. dentata*, *H. hemerocallidea*, *C. asiatica*, and *C. contracta* all demonstrated strong radical scavenging potentials. The activity of *N. stellata* stems was moderate while *P. viridiflorum* showed weak activity, respectively. On the other hand, ABTS ^+^ assay results revealed that *A. melegueta* and *C. dentata* have very strong activity, greater than those of *N. stellata* (flowers and leaves), H. *hemerocallidea*, *C. asiatica*, and *C. contracta*, all of which are classified to have strong antioxidant activity values. The results obtained from both qualitative antioxidant and quantitative antioxidant assays confirm that *A. melegueta* proved to possess the strongest radical scavenging capacity, stronger than that of ascorbic acid and Trolox standards using DPPH assay and compatible to those obtained with ABTS^+^. Similarly, Onoja et al. [42] observed potent antioxidant activity *in vitro* antioxidant assay of *A. melegueta* seed extract. Flowers and leaves of *N. stellata* and *C. dentata* also exhibited very strong antioxidant activity for both DPPH and ABTS^+^, the strong antioxidant activity reported in this study was agreeable with those reported by Oyedemi et al. [43] for *C. dentata and N. stellata*, respectively. *C. asiatica* showed activity within the strong antioxidant activity range 10–50 μg/mL. Similarly, Kumari et al. [44] and Rahman et al. [45] reported values within this range in ethanolic extracts. By comparing the antioxidant spectrometric methods used in this study, it can be inferred that the quantified antioxidant activity was not equivalent and that ABTS^+^ method displayed a lower sensitivity compared to the DPPH method. The results obtained using the TLC bioautography method did somewhat depict the findings observed in the spectrophotometric serial dilution methods. The presence of phenolic compounds found in different plant species may have contributed to various levels of antioxidant activities observed.

The methanolic plant extracts were further investigated for their detoxification abilities against AFB_1_ over time. The *C. dentata* bark extract significantly reduced AFB_1_ followed by *C. asiatica* and *A. melegueta* after 48 h. The detoxification capabilities of *C. dentata* may be attributed to the phenolic compounds it possessed. This study revealed that the extract possessed the highest phenolic content and showed very good antioxidant activity. The bark is used to treat cancer, hypertension, obesity, sexually transmitted infections, and stomach issues [46]. A similar study by Al-Owaisi et al. [11] on aqueous *C. asiatica* extract obtained over 70 % detoxification activity against AFB_1_. Iram et al. [9] established that the reduction in toxicity of AFB_1_ by *Ocimum basilicum* and *Cassia fistula* plant extracts was linked to removal of the double bonds in the terminal furan ring and lactone ring modification. This is interesting because the double bond in the terminal furan ring of AFB_1_ molecules have been linked to its hazardous and carcinogenic properties [47]. Literature shows that the longer AFBs are incubated with plant extracts, the more modifications to their molecular structures occur, thus lowering their toxicity [7,8]. This study demonstrated a time-dependent decrease in AFB_1_ levels by all tested plant extracts. This result is in line with the study by Iram et al. [9] and Ponzilacqua et al. [10], whereby a rise in AFB_1_ degradation with increasing incubation times was observed for the different plant extracts. The differences in phenolic and flavonoid content as well as the antioxidant potentials of various extracts may explain the differences in the detoxification capabilities of the plant extracts studied and those previously studied and published in the literature [[7], [8], [9], [10], [11]].

The detoxification activities of *C. contracta*, *N. stellata* stems and *P. viridiflorum* were undefined due to matrix effect, which may be a result of ion suppression or enhancement. According to Parham [48] different solutions can be used to reduce or remove matrix effects. These include enhancing extraction and clean-up processes, switching to a different type of ionization, employing corrective calibration methods, and the use of an internal standard. It was noted that the incubation temperature had a significant impact on AFB_1_ as over 50 % of the toxin was degraded in the controls. Iram and co-workers reported temperature degradation of 10.40 % in AFB_1_+H_2_O control and linked this degradation to the synergistic effect of heat and moisture [9]. However, this value is significantly lower than those obtained in this study. The stability of the AFB_1_ standard used and the type of control solvent used together with the synergistic effect of heat and moisture could have contributed to this.

## 5 Conclusion

The plant extracts in this study revealed the presence of numerous phytochemicals. However, methanol extracts of *C. dentata* bark, *C. asiatica* leaves, and *A. melegueta* seeds possessed high TPC, TFC with high antioxidant activities and greater detoxifying potentials against AFB_1_ in this study. This suggests that they could be used as natural AFB_1_ detoxifying agents in the agriculture and food industries. It can therefore follow that plant extracts with higher TPC, TFC, and antioxidant potential would exhibit better activity against AFB_1_ degradation. Findings from the research demonstrated that the abundance of bioactive compounds with antioxidant activity plays a role in AFB_1_ detoxification activity.

## Funding

The 10.13039/501100001321National Research Foundation South Africa (NRF Postgraduate scholarship, MND190616448060); DSI/NRF innovation Postdoctoral scholarship (PDG190211415413) and the 10.13039/501100006565University of Johannesburg, South Africa supported the work.

## Data availability

Data will be made available on request.

## CRediT authorship contribution statement

**Mavie Rose Kongolo Kalemba:** Writing – original draft, Methodology, Investigation, Formal analysis, Data curation. **Rhulani Makhuvele:** Writing – review & editing, Supervision, Project administration, Funding acquisition, Conceptualization. **Patrick Berka Njobeh:** Writing – review & editing, Supervision, Resources, Funding acquisition, Conceptualization.

## Declaration of competing interest

The authors declare the following financial interests/personal relationships which may be considered as potential competing interests:Mavie Rose Kongolo Kalemba and Rhulani Makhuvele reports financial support was provided by 10.13039/501100001321National Research Foundation. If there are other authors, they declare that they have no known competing financial interests or personal relationships that could have appeared to influence the work reported in this paper.

## Lists of abbreviations

ABTS: 2,2′-azino-bis (3-ethylbenzothiazoline-6-sulfonic Acid)
AFB_1_: Aflatoxin B_1_
AFBO: AFB1-8-9-epoxide
AlCl_3_: Aluminum chloride
BEA: Benzene/ethanol/ammonium hydroxide (90:10:1)
CEF: Chloroform/ethyl acetate/formic acid (5:4:1)
DL: Desolation line
DPPH: 2,2-diphenyl-1-picrylhydrazyl
EMW: Ethyl acetate/methanol/water (40:5.4:4)
ESI: Electron spray ionization
GAE: Gallic acid equivalents
MRM: Multiple reaction monitoring
TFC: Total flavonoid content
TLC: Thin layer chromatography
TPC: Total phenolic content
TQD: Triple quadrupole
ROS: Reactive oxygen species
LC-MS: Liquid chromatography mass spectrometry
MS: Mass spectrometry
QE: Quercetin equivalent
UV: Ultraviolet
